# More complications in uncemented compared to cemented hemiarthroplasty for displaced femoral neck fractures: a randomized controlled trial of 201 patients, with one year follow-up

**DOI:** 10.1186/s12891-017-1526-0

**Published:** 2017-04-21

**Authors:** Sophie Moerman, Nina M. C. Mathijssen, Dieu D. Niesten, Roeland Riedijk, Willard J. Rijnberg, Sander Koëter, Keetie Kremers van de Hei, Wim E. Tuinebreier, Tim L. Molenaar, Rob G. H. H. Nelissen, Anne J. H. Vochteloo

**Affiliations:** 1 Department of Orthopaedics, Reinier de Graaf Group, Reinier de Graafweg 5, 2625 AD Delft, The Netherlands; 2 Tergooi hospital, Hilversum, The Netherlands; 3 Rijnstate Hospital, Arnhem, The Netherlands; 4 Canisius Wilhelmina Hospital, Nijmegen, The Netherlands; 5 Department of Surgery/Traumatology, Erasmus MC, Rotterdam, The Netherlands; 6 Leiden University Medical Center, Leiden, The Netherlands; 7 Centre for Orthopedic Surgery OCON, Hengelo, The Netherlands

**Keywords:** Hip fracture, Arthroplasty, Bone cement, Complication

## Abstract

**Background:**

It is unclear whether cemented or uncemented hemiarthroplasty is the best treatment option in elderly patients with displaced femoral neck fractures. Previous randomized trials comparing cemented and uncemented hemiarthroplasty have conflicting results. We conducted a randomized controlled trial to compare cemented and uncemented hemiarthroplasty.

**Methods:**

This multicenter parallel-randomized controlled trial included patients of 70 years and older with a displaced femoral neck fracture (Garden type III or IV). Inclusion was between August 2008 and June 2012. Patients were randomized between a cemented hemiarthroplasty, type Müller Straight Stem or an uncemented hemiarthroplasty, type DB-10. Primary outcomes were complications, operation time, functional outcome (measured by Timed-Up-and-Go (TUG) and Groningen Activity Restriction Scale (GARS)) and mid-thigh pain. Health Related Quality of Life (HRQoL, expressed with the SF-12) was measured as an secondary outcome. Follow up was 1 year.

**Results:**

In total 201 patients were included in the study (91 uncemented, 110 cemented hemiarthroplasties) The uncemented group showed more major local complications (intra- and postoperative fractures and dislocations) odds ratio (95% confidence interval) 3.36 (1.40 to 8.11). There was no difference in mean operation time (57.3 vs 55.4 min). There were no differences in functional outcomes (TUG 12.8 (9.4) vs. 13.9 (9.0), GARS 43.2 (19.7) vs. 39.2 (16.5)) and mid-thigh pain (18.6 vs 21.6%). Physical component SF-12 HRQoLwas lower in the uncemented group (30.3 vs. 35.3 *p* < 0.05 after six weeks, 33.8 vs 38.5 *p* < 0.05 after 12 weeks).

**Conclusion:**

A cemented hemiarthroplasty in elderly patients with a displaced femoral neck fracture results in less complications compared to an uncemented hemiarthroplasty.

**Trial registration:**

Netherlands Trial Registry; NTR 1508, accepted date 27 okt 2008

**Electronic supplementary material:**

The online version of this article (doi:10.1186/s12891-017-1526-0) contains supplementary material, which is available to authorized users.

## Background

Hip fractures are a rising problem in our aging society. An increase in the incidence of hip fractures in Europe from 615.000 in 2010 to 815.000 in 2025 (+32%) due to demographic changes is expected [1]. Elderly patients with a dislocated femoral neck can be treated effectively with hemiarthroplasty [2]. However, there is a persistent controversy regarding the use of cement [3].

In cemented hemiarthroplasties, polymethylmethacrylate bone cement is used during surgery to create a solid bone-implant interface. A potential advantage of cement is less post-operative mid-thigh pain, as the hemiarthroplasty is more firmly fixed within the femur [4]. A potential negative effect of using cement is the Bone Cement Implantation Syndrome (BCIS), characterized by hypoxia and/or hypotension in combination with an unexpected loss of consciousness which occasionally occurs following cement insertion [5]. This complication may be fatal.

Uncemented hemiarthroplasties are placed press-fit in the femur. In the weeks after the surgery the bond between femur and the stem is dependent on osseous integration [6]. However, bone quality is generally poor in elderly hip fracture patients, which may lead to periprosthetic fractures during press-fit placement or inadequate bony in-growth post-operatively [7].

Both NICE and AAOS guidelines advise to use cemented implants [2, 8]. However, despite these guidelines, database studies show that 22 to 34% of the hemiarthroplasties are used without cement [9, 10].

The Cochrane review of 2011 included six trials comparing cemented and uncemented hemiarthroplasty and demonstrated a reduction of the amount of postoperative pain, an improvement in postoperative function and less implant-related complications when cement was used, but a longer operation time. There was no difference in adverse events or mortality [3]. After this review three more randomized trials were published. One found no difference in functional outcome, complications and mortality [11]. Another found more complications (subsidence, intraoperative fracture and postoperative fracture) in the uncemented group, with no differences in pain or mortality [12]. The third trial found better functional outcomes and less intraoperative fractures in the cemented group [13]. Thus the controversy whether to use an cemented or uncemented hemi arthroplasty in the older patients with a displaced femoral neck fracture persists.

Therefore, we compared uncemented and cemented hemiartroplasties in a parallel randomized controlled trial. We hypothesized that an uncemented hemiarthroplasty for a displaced femoral neck fracture in elderly patients would have at least comparable radiological and functional outcomes and complication rate as a cemented hemiarthroplasty and that non-cementing of hemiarthroplasty would result in a shorter operation time [14].

## Methods

This multicenter parallel randomized controlled trial included patients with a displaced femoral neck fracture. The study was approved by the Regional Ethics Committee (NL19200.098.07, METC07 –118). The trial was registered in the Netherlands Trial Registry NTR 1508 (http://www.trialregister.nl). The protocol was published before start of the study [14].

All patients were admitted to one of the participating hospitals (Reinier de Graaf hospital, Delft; Rijnstate hospital, Arnhem and Canisius Wilhelmina hospital, Nijmegen), large district hospitals in the Netherlands. Inclusion was between August 2008 and June 2012. Included were patients aged 70 years or older, with a displaced femoral neck fracture (Garden type III or IV) suitable for hemiarthroplasty. Excluded were patients with a pathological fracture, a fracture older than seven days or ASA-IV or V classification. Orthopedic residents, trained for this study, performed inclusion.

**Fig. 1:**
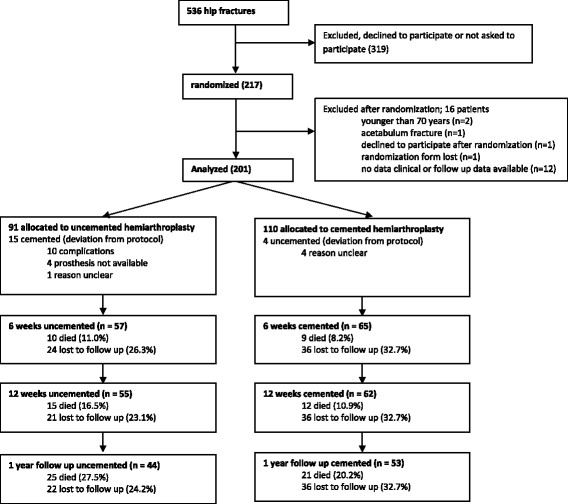
Flowchart of the recruitment and flow of patients with femoral neck fractures during the study

All patients gave informed consent. In case of (mental) incompetence of the patient, his or her legal representative was consulted to obtain informed consent. Patients were randomized following a simple randomization procedure in the operation theatre by the orthopedic surgeon through opaque sealed envelopes. These were prepared by A.J.V. and kept at the operation theatre of each of the three hospitals. 200 opaque sealed envelopes were prepared. However, 16 patients could not be included in our trial due to variable reasons (Fig. 1), which forced us to prepare another 16 envelopes.

The patients were blinded for the type hemiarthroplasty they received, although we acknowledge the possibility that they might be able to tell after seeing their radiographs during the outpatient clinic visits. Surgeons and outcome assessors were aware of the allocated arm.

Patients received a cemented hemiarthroplasty, type Müller Straight Stem (Zimmer - Biomet, 1800 West Center St. Warsaw, Indiana, USA) or an uncemented hemiarthroplasty, type DB-10 (Zimmer- Biomet, 1800 West Center St. Warsaw, Indiana, USA). The cemented hemiarthroplasty, the Muller straight stem has a small proximal collar and two longitudinal grooves to enable good cement adhesion. The non-cemented DB-10 is a straight collared stem with metaphyseal anchoring and on the surface full hydroxyapatite coating on macro-structured titanium and grooves. If complications occurred during the procedure, the surgeon could change the procedure to ensure best medical practice. Operating technique was according to the manufacturer instruction. In the participating hospitals there was experience with both cemented and uncemented hip arthroplasty. Either an orthopedic surgeon or registrar performed the operation. Cementing technique involved vacuum mixing, cement plug, saline pulsed lavage and retrograde introduction of cement with a cement gun. The approach was up to the surgeon’s preference, as Parker’s Cochrane analysis has shown that insufficient evidence is available for superiority of either approach [3].

Each patient received physiotherapy therapy, analgesia and trombo-embolic prophylaxis according to the protocol of the hospital in which they were treated.

Preoperatively, social demographic data (age, sex, place of residence), ASA— (American Society of Anesthesiologists) classification [15], Body Mass Index (BMI), Minimal Mental State Examination (MMSE) [16] were obtained. Patients were asked to score their pre-fracture mobility and Health Related Quality of Life (HRQoL) using the New Mobility Score (NMS) [17], Groningen Activity Restriction Scale (GARS) [18] and the SF-12 [19]. Patients were asked if they mobilized with an aid indoors and outdoors with or without aid and whether they received homecare. The baseline hemoglobin level was measured. The surgical approach, the type of surgeon (consultant or registrar) and kind of anesthesia were registered.

Outcomes measured during operation were operation time (defined as skin-to-skin surgical time, measured in minutes) and blood loss (in centiliter, estimated by the surgeon).

Length of stay, decrease in hemoglobin level and transfusion rate were measured postoperatively.

All patients were invited for follow up at six, 12 and 52 weeks postoperatively. When the patient was not able to visit the outpatient clinic, the questionnaires were mailed to the patient or its relatives. During follow-up functional outcome was measured using Timed-Up and- Go (TUG) score [20], GARS [18] and NMS [17]. HRQoL, expressed in the SF-12 [19], was measured. The SF-12 was divided in a Physical Component summary Score (PCS) and a Mental Component summary Score (MCS). Mid-thigh pain (defined as pain explicitly in the front and mid part of the femur) pain and place of residence were registered. Complications during surgery, hospital stay and the year thereafter were recorded. The complications were defined and ranked in the modified Elixhauser mode, as described by Parvizi [21]. Mortality was scored meticulously by repeated consultation of the population registers of the counties in the region of the hospital as well as the hospital’s patient registration systems for the full length of follow-up.

A radiograph was obtained on the first postoperative day and after six weeks, 12 weeks and 1 year. Adequate positioning of the stem was defined as within 10° varus or valgus position with respect to the femoral axis. Fissures, fractures, subsidence and loosening were noted.

### Analysis

Primary outcomes were complications, operation time, functional outcome and post-operative mid-thigh pain. A Bonferroni correction was applied for the eight primary outcome measures (4 types of complications, operation time, GARS, TUG and mid-thigh pain at 1 year) making *p* < 0.006 significant. Secondary outcomes were return to place of residence as percentage of pre-fracture situation, HRQoL and adequate radiological positioning of the hemiarthroplasty [14].

### Determination of sample size

The complete power calculation is published in our protocol [14] We expected (based on the literature in 2008) that midthigh pain in uncemented prosthesis would be 30% and in cemented prosthesis 7.5%.

$$ \uppi 1 = 30\%,\ \uppi\ 2 = 7.5\%,\ \uppi = \left(30\% + 7.5\%\right)/2 = 18.75\% $$

$$ \mathrm{n}1=\mathrm{n}2\ \ge\ 21 \ast \left(0,1875\ast \left(1{\textstyle \hbox{-} }0,1875\right)\right)/\left(0,225\right)2=63.2. $$

While we expected 25% 1-year mortality and 10% lost-to follow-up we raised this number by 35%. Thus a total of 86 patients a group were needed. The calculations for the other three primary outcome measures (duration of surgery, functional outcome and complications) produced lower patients numbers [14]. From a practical point of view we choose a total of 100 patient per group.

### Statistical analysis

All analyses were performed using SPSS software (SPSS Inc., Chicago, IL, USA). The differences in outcome measures were analyzed using an independent sample student *T*-test (for continuous data) and Chi-Square Test (for categorical data), setting the level of significance at *p* < 0.05 for secondary outcomes. All outcomes analyses were done twice: both for as treated analysis and for intention to treat. The numbers given in the results section represent the intention to treat analysis. We will report explicitly if differences exist between as treated analysis and intention to treat analysis.

## Results

In total 201 patients were analyzed (Fig. 1) 91. Were randomized to an uncemented, 110 to a cemented hemiarthroplasty. In 15 of the 91 (16%) patients randomized to an uncemented hemiarthroplasty a cemented hemiarthroplasty was used instead. In ten patients this was due to intraoperative complications (i.e. fracture of the femur). In four patients the necessary instruments or prosthesis were not present and in one patient the reason was unknown. Four of the 110 (4%) patients randomized to a cemented hemiarthroplasty received an uncemented hemiarthroplasty. In none of these cases the reason for this breach of the protocol was clear.

Table 1 shows baseline characteristics of both groups.

**Table 1:** Baseline characteristics

	Uncemented (91)	Cemented (110)
Age (mean (SD))	84.0 in 91 (6.7)	83.0 in 110 (6.2)
Sex female (number, %)	61 out of 91 (67%)	82 out of 110 (75%)
ASA classification (number, %)		
I	7 out of 91 (8%)	6 out of 110 (6%)
II	51 out of 91 (56%)	71 out of 110 (65%)
III	33 out of 91 (37%)	33 out of 110 (30%)
BMI (mean (SD))	24.3 in 60 (3.5)	24.1 in 73 (3.4)
MMSE < 24 (number, %)	15 out of 44 (34%)	23 out of 56 (41%)
Mobile without aid indoors (number, %)	32 out of 73 (44%)	41 out of 81 (51%)
Mobile without aid outdoors (number, %)	21 out of 73 (29%)	32 out of 81 (40%)
NMS (mean (SD))	5.2 in 71 (2.7)	5.5 in 77 (3.0)
GARS (mean (SD))	41.1 in 71 (16.8)	41.7 in 78 (18.6)
SF-12, Physical Component (mean (SD))	37.1 in 65 (11.2)	37.9 in 65 (12.3)
SF-12, Mental Component (mean (SD))	46.8 in 65 (10.9)	48.3 in 65 (12.1)
Living at home (number, %)	52 out of 73 (71%)	58 out of 84 (69%)
No domestic or homecare (number, %)	37 out of 64 (58%)	39 out of 75 (52%)
Hemoglobin level (g/dL) (mean (SD))	12.8 in 91 (1.5)	12.7 in 110 (1.8)
Surgical approach (number, %)		
Straight lateral	41 out of 90 (46%)	49 out of 110 (45%)
Postero lateral	45 out of 90 (50%)	61 out of 110 (56%)
Anterior	4 out of 90 (5%)	4 out of 110 (4%)
Consultant (vs. registrar) (number, %)	24 out of 91 (26%)	43 out of 110 (39%)
Spinal anesthesia (vs. general) (number, %)	68 out of 90 (76%)	80 out of 107 (75%)

### Primary outcomes

#### Complications

The 1-year complication rate per category as categorized by Parvizi is shown in Table 2 [21]. Major local complications were more frequent in the uncemented hemiarthroplasty group; (odds ratio; 95% CI) (3.36; 1.40 to 8.11). In the uncemented group there were 14 periprostetic fractures. 12 were noticed perioperative, in ten of these patients the procedure was converted to a cemented procedure, in two patients a cerclage wire was used. In two patients of the uncemented group and 3 of the cemented group a fracture was noted postoperative, these patients were treated with protected weight baring. Analysis according the as treated analysis approach (instead of intention to treat) showed no differences between cemented and uncemented hemiarthroplasty regarding major local complications. Minor local complications (0.73; 0.33 to 1.59), major systemic (1.31; 0.71 to 2.41) and minor systemic complications (0.96; 0.47 to 1.93) were comparable between groups. The 1-year mortality rate was higher in the uncemented group (25 (27.4%)) compared to the cemented group (21 (19.0%)) but did not reach significance (*p* = 0.18). One major systemic complication was a patient who died just after injecting the cement into the femoral canal, potentially caused by BCIS, however autopsy was not performed.

**Table 2:** One-year complication rate per category as categorized by Parvizi

		Uncemented (91)	Cemented (110)	*P*
Major systemic	Death	25	21	(0.18)
	Tachyarrhythmia	1	4	
	Myocardial infarction	4	2	
	Pulmonary embolus	1	6	
	Acute renal failure	3	2	
	Stroke and/or TIA	3	3	
	Bowel obstruction	0	1	
	Total number of patients with >/=1 major systemic complication ^a^	29 out of 91 (31.9%)	29 out of 110 (26.4%)	0.41
Minor systemic	Anemia	30	39	
	Urinary tract infection	14	22	
	Mental status change	23	21	
	Gastric hypomotility	0	2	
	Deep venous thrombosis	0	1	
	Pneumonia	14	12	
	Social complication	2	9	
	Others	2	2	
	Total number of patients with >/=1 minor systemic ^a^	73 out of 91 (80.2%)	89 out of 110 (80.9%)	0.92
Major local	Peripheral nerve injury	0	1	
	Infection leading to revision	0	1	
	Periprosthetic fracture	14	3	
	*intraoperatively*	*12*	*0*	
	*postoperatively*	*2*	*3*	
	Dislocation	5	3	
	Total number of patients with >/= 1 major local complication ^a^	19 out of 91 (20.9%)	8 out of 110 (7.3%)	0.005
Minor local	Hematoma	1	6	
	Persistent wound drainage	3	4	
	Superficial wound infection	3	6	
	Skin blisters	1	1	
	Other	6	2	
	Total number of patients with >/= 1 minor local complication ^a^	12 out of 91 (13.2%)	19 out of 110 (10.9%)	0.42

^a^The number of patients with a complication in a category is not equal to the sum of complications in a category, while some patients had more than 1 complication

#### Operation time

The mean (95% CI) operation time was comparable between uncemented and cemented hemiarthroplasty: 57.3 min (52.8–61.9) and 55.4 min (52.0–58.9) respectively.

#### Functional outcome

At no point of follow-up a difference was found in functional outcome, expressed in the TUG and GARS score (Table 3). The pre-defined clinically relevant worsening from 30 to 42 of the TUG was not met in a single patient in one of the groups. TUG was poorly registered (53% at six weeks, 51% at 12 weeks, 48% at 1 year, corrected for mortality).

**Table 3:** Functional outcome measures at six, 12 weeks and 1 year and radiological outcome post-operative and any time during follow up

		Uncemented			Cemented			
		Mean (SD)	97.5% CI	Number	Mean (SD)	97.5% CI	Number	*P*
Timed up and go	6 weeks	18.7 (13.8)	13.9–23.5	45	18.7 (12.9)	14.6–22.9	51	0.99
	12 weeks	16.2 (12.4)	11.5–20.9	38	15.5 (8.5)	12.7–18.2	50	0.74
	1 year	12.8 (9.4)	8.9–16.7	33	13.9 (9.0)	10.1–16.7	41	0.79
GARS* (iADL)	6 weeks	53.1 (14.9)	48.5–57.8	54	50.0 (15.3)	45.7–54.4	65	0.27
	12 weeks	45.7 (17.0)	40.3–51.2	52	45.3 (16.6)	40.4–50.1	62	0.88
	1 year	43.2 (19.7)	36.2–50.2	43	39.2 (16.5)	34.0–44.4	53	0.28
NMS	6 weeks	3.7 (2.5)	2.9–4.4	53	3.5 (2.4)	2.8–4.1	64	0.65
	12 weeks	4.5 (2.8)	3.6–5.4	51	4.8 (3.1)	3.8–5.7	59	0.68
	1 year	4.7 (3.2)	3.6–5.8	44	5.7 (2.9)	4.8–6.7	50	0.12
SF-12 Physical component	**6 weeks**	30.3 (6.9)*	27.9–32.6*	**47**	35.3 (9.3)*	32.4–38.2*	**54**	**0.003**
	**12 weeks**	33.8 (9.8)*	30.6–37.1*	**48**	38.5 (9.9)*	35.4–41.6*	**54**	**0.018**
	1 year	36.8 (10.7)	32.9–40.8	40	37.5 (9.4)	34.3–40.7	50	0.76
SF-12 Mental component	6 weeks	45.0 (13.0)	40.7–49.5	47	47.4 (11.0)	44.0–50.8	54	0.33
	12 weeks	47.7 (11.2)	43.9–51.4	48	49.5 (11.0)	46.0–52.9	54	0.41
	1 year	49.3 (11.2)	45.2–53.4	40	51.4 (10.1)	47.9–54.9	50	0.36
		Number (%)			Number (%)			
Mid-thigh pain	6 weeks	23 out of 55 (42%)			20 out of 63 (32%)			0.26
	12 weeks	19 out of 55 (35%)			17 out of 61 (27%)			0.83
	1 year	8 out of 43 (19%)			11 out of 51 (22%)			0.72
Varus or valgus deviation	Post operative	8 out of 89 (9%)			7 out of 107 (7%)			0.76

*p < 0.05

* *P*<0.05 The NMS was at all moments of follow-up comparable (Table 3).

#### Post-operative mid-thigh pain

There was no difference in post-operative mid-thigh pain between both groups at any time during follow up. It was present in 43 patients (36%) after six weeks, which decreased to 31% after 12 weeks and 20% after 1 year (Table 3).

### Secondary outcomes;

There was no difference in the number of patients who returned to their baseline place of residence after 1 year (28 patients (72%) vs. 37 patients (80%) *p* = 0.88).

**Fig. 2:**
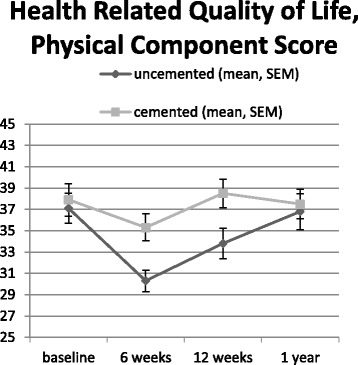
Health related quality of life, physical component score

The SF-12 MCS did not differ between the cemented and the uncemented group. (Table 3) However, the SF-12 PCS was lower at six and 12 weeks postoperatively in the uncemented hemiarthroplasty group. This difference resolved after 1 year (Table 3 and Fig. 2). Analyzing the results according the as treated analysis showed a lower PCS for uncemented hemiarthroplasty at six weeks (30.3 vs. 34.8 *p* = 0.01), and a difference at 12 weeks (34.0 vs. 37.9) which nearly did reach significance (*p* = 0.056). There was no difference at 1 year after surgery (36.6 vs.37.6 *p* = 0.65).

Radiographs were taken direct post-operative and after 6 and 12 weeks and 1 year. Five patients deceased before the post-operative radiograph was obtained. Eight varus or valgus deviations in the uncemented group and seven in the cemented group (*p* = 0.76) were found on the post-operative radiograph. Loosening or subsidence was observed in 13 (20%) of the uncemented and five (6%) of the cemented hemiarthroplasties (*p* = 0.007) any time during follow up. Four (2%) revision operations were performed: three due to loosening (all in the uncemented group) and one for infection (in the cemented group) (*p* = 0.162).

There were no differences in length of stay between both groups (mean 11 (SD 7.7) days uncemented vs. 11 days (SD 8.3) cemented *p* = 0.83), loss in hemoglobin level (g/dl) after surgery (uncemented mean 2.2 (SD 1.4) vs. cemented mean 2.0 (SD 1.5) *p* = 0.31) and transfusion rate (uncemented 17 (24%) vs. cemented 22 (26%) *p* = 0.74). Surgeon estimated blood loss was larger in the uncemented group (mean 288 mL (sd 213) vs. mean 220 mL (sd 143) *p* = 0.03) (Additional file 1: Table S1).

## Discussion

The most important finding of our study was that major local complications were more frequent (odds ratio; 95% CI) (3.36; 1.40 to 8.11) in the uncemented hemiarthroplasty group compared to the cemented group. In elderly patients with a displaced femoral neck fracture hemiarthroplasty is a widely accepted treatment of choice [2]. Previous randomized trials comparing cemented and uncemented hemiarthroplasty give conflicting results on this [4, 11–13, 22].

A periprosthetic fracture was the most common major local complication in the uncemented group (15%). Previous papers comparing fracture rate are heterogenic. Some studies found a higher fracture rate in the uncemented group, ranging from 5.5 to 12% [3, 12, 13, 23] whereas others demonstrated no difference in fracture rate [4, 11, 22]. The fracture rate in the current study is higher than previous papers demonstrate. The teaching of registrars might have attributed to this. This can be due to the design of the DB-10 stem (proximal fitting) compared to the stems used in the other papers. Furthermore, teaching of registrars might have attributed to this as well: the level of experience of the operation surgeon is not always mentioned, but for example in the studies of Inngul, DeAngelis and Parker the operations were always performed by consultant orthopaedic surgeons [4, 11, 13]. However this high complication rate might better reflect the everyday practice with registrars often performing this type of operations.

One-year mortality rate was higher in the uncemented group (25 (27.4%) compared to the cemented group 21 (19.0%) but did not reach significance (*p* = 0.16). This is in contrast to other randomized controlled trials [4]. The register studies show higher mortality in the first operative days in cemented hemi arthroplasty [24–26]. However the Australian and British register shows lower mortality in cemented hemiarthroplasty the year thereafter [10, 27]. Power analysis in our study was not performed on finding differences in mortality. In our trial one patient died intraoperatively, probably due to Bone Cement Implantation Syndrome (BCIS). BCIS is a major side effect of cement implantation, it has no agreed definition but is characterized by a number of clinical features with amongst others hypoxia, hypotension, cardiac arrhythmias, increased pulmonary vascular resistance and cardio-respiratory collapse [5]. Guidelines to minimize the risk for BCIS by both surgeon and anesthetist are recently published [28].

In contrast to our hypothesis and literature, we did not find a difference in operation time between groups [3, 22]. Intraoperative complications in the uncemented group might have affected this, however equality in the mean operation time persisted when we analyzed our data in an As Treated analysis. Teaching residents during might have affected the operation time in such way that the difference disappeared.

Functional outcome (GARS score and TUG test) was not different between both groups. However TUG was measured only in 53% of all patients at 6 weeks, 51% at 12 weeks and 48% at 1 year. Probably TUG is not a very useful outcome measure in this frail population as mobility was too poor to measure well. Mobility expressed as the NMS was comparable between the two groups. In literature different outcome measures for functional outcome have been used (Oxford or Harris Hip Score [12, 13, 22] Older Americans Resources and Services Instrument [11] Barthel Index [22]) A meta-analysis pooled results of five trials (491 patients) and found that patients with an cemented hemiarthroplasty had a better hip function after 1 year [7].

Mid-thigh pain is known to be more prevalent in uncemented prostheses, however the reported incidence differs tremendously [4, 7, 29]. In our study presence of mid-thigh pain was comparable between groups. Several factors can be of influence on post-operative mid-thigh pain such as sizing, design and stiffness of a prosthesis [29].

Radiological follow up showed loosening or subsidence in 13 uncemented hemiarthroplasty, which led to a revision in three cases. Subsidence in uncemented (hemi) arthroplasty is a common finding; one trial found a larger subsidence in the uncemented hemiarthroplasty [12]. In The Norwegian Hip Fracture Register patients treated with an uncemented hemiartroplasty had a 2.1 times increased risk of revision compared with patients treated cemented prostheses. This increased risk of re-operation was due to peri-prosthetic fracture HRR 17 and aseptic loosening HHR 17 [30]. A combined analysis of the Norwegian and Swedish registers (33.205 hip fractures in patients older than 60 years treated with hemiarthroplasty) also found more reoperations in uncemented stems (HR2.2) [9].

PCS of HRQoL was lower in patients treated with an uncemented hemiarthroplasty at six weeks and three months after surgery. PCS HRQoL is known to decrease in the first three months and recover thereafter [31]. The larger complication rate might have led to this lower PCS HRQoL, although we would have expected to find a difference in functional and mobility scores as well. One previous trial found higher HRQoL (expressed in EQ5days) in the cemented group [13] at 4 and 12 months, another trial did not [22]. The latter did not find a difference in complications either [22]. HRQoL was an secondary outcome in this trial, thus no power calculations were made and dropout at follow-up was quite high: therefore the difference we found might be due to coincidence and has to be verified in further trials.

The large number of patients, the randomized design and outcome measures on both functional and radiological outcomes make the current study worthwhile. Furthermore, we did not exclude patients with cognitive disorders. The latter makes our study generalizable to all elderly hip fracture patients treated with hemiarthroplasty.

However, our study does have limitations. First, many (293) patients (or their caretakers) declined to participate in our study or were not asked to participate, thus selection bias might be present. Second, poor registration has led to incompleteness of some of the baseline data. This led to the exclusion of 12 patients after randomization because of missing baseline data, 5 patients were excluded after randomiz ation due to other reasons (Fig. 1). Deviation from the protocol occurred in 19 patients (9% of analyzed cases). Therefore both as treated analysis and intention to treat were performed. More deviations from protocol were present in the uncemented group (15 vs. 4) which might have caused a bias.

Furthermore, we had a substantial percentage (24%) of patients who were lost to follow-up. This might be due to the inclusion of patients with cognitive disorders (38% had an MMSE less than 24) and high age of the participators, resulting in their caretakers to refrain from extra stress by those patients by filling in follow-up forms.

This trial adds value to the discussion whether to use a cemented or an uncemented hemiartroplasty in femoral neck fractures. Conflicting evidence on this matter is published the last few years [4, 11–13, 22]. Trial design, prosthesis design, inclusion criteria and whether the trail was performed in a teaching hospital or not might all have been of influence of these published results.

## Conclusions

Elderly patients with a displaced femoral neck fracture treated with an uncemented hemiarthroplasty had more periprosthetic fractures, loosening, reoperations and lower quality of life compared to patients with a cemented stem. Operation time, functional outcome and mid-thigh pain were comparable between groups. Based on these findings, and earlier work [3, 12, 13] we conclude that in elderly patients with a displaced femoral neck fracture a cemented hemiarthroplasty is favorable compared to an uncemented stem.

## Additional file

Additional file 1: Table S1. Perioperative details of uncemented and cemented hemiarthroplasty. length of stay, loss in hemoglobin, estimated blood loss and transfusion rate of uncemented and cemented hemiarthroplasty (DOCX 11 kb)

## Abbreviations

ASA -classification: American Society of Anesthesiologists -classification
BCIS: Bone cement implantation syndrome
BMI: Body mass index
GARS: Groningen activity restriction scale
HRQoL: Health related quality of life
MCS: Mental component summary score
MMSE: Minimal mental state examination
PCS: Physical component summary score
TUG: Timed-up-and-go
