# Erratum to: more complications in uncemented compared to cemented hemiarthroplasty for displaced femoral neck fractures: a randomized controlled trial of 201 patients, with one year follow-up

**DOI:** 10.1186/s12891-017-1661-7

**Published:** 2017-07-17

**Authors:** Sophie Moerman, Nina M. C. Mathijssen, Dieu D. Niesten, Roeland Riedijk, Willard J. Rijnberg, Sander Koëter, Keetie Kremers van de Hei, Wim E. Tuinebreijer, Tim L. Molenaar, Rob G. H. H. Nelissen, Anne J. H. Vochteloo

**Affiliations:** 1 Department of Orthopaedics, Reinier de Graaf Group, Reinier de Graafweg 5, 2625 AD Delft, The Netherlands; 2 Tergooi hospital, Hilversum, The Netherlands; 3 Rijnstate Hospital, Arnhem, The Netherlands; 4 Canisius Wilhelmina Hospital, Nijmegen, The Netherlands; 5 Department of Surgery/Traumatology, Erasmus MC, Rotterdam, The Netherlands; 6 Leiden University Medical Center, Leiden, The Netherlands; 7 Centre for Orthopaedic Surgery OCON, Hengelo, The Netherlands

## Erratum

After publication of this article [1] it came to our attention that the author Wim E. Tuinebreijer was incorrectly included as Wim E. Tuinebreier. The correct spelling of this author name is included in this erratum and updated in the original article.
