# Supplementary material for: More complications in uncemented compared to cemented hemiarthroplasty for displaced femoral neck fractures: a randomized controlled trial of 201 patients, with one year follow-up
Source: BMC Musculoskelet Disord. 2017 Apr 21;18:169. doi: 10.1186/s12891-017-1526-0 (PMC5399836; doi:10.1186/s12891-017-1526-0)
Supplement: Additional file 1: Table S1. — Perioperative details of uncemented and cemented hemiarthroplasty. length of stay, loss in hemoglobin, estimated blood loss and transfusion rate of uncemented and cemented hemiarthroplasty (DOCX 11 kb) [file 12891_2017_1526_MOESM1_ESM.docx]

|  | **uncemented** | | **cemented** | |  |
| --- | --- | --- | --- | --- | --- |
|  | **Mean (SD)** | **N** | **Mean (SD)** | **N** | **p** |
| Length of stay (days) | 10.51 (7.6) | 91 | 10.76 (8.33) | 110 | 0.83 |
| Loss in hemoglobin level (g/dL) | 2.20 (1.35) | 91 | 1.98 (1.54) | 109 | 0.31 |
| Estimated blood los (mL) | **288 (213)** | **71** | **220 (143)** | **73** | **0.027** |
|  | Number (%) | | Number (%) | |  |
| Transfusion rate | 17 out of 72 (23.6%) | | 22 out of 85 (25.9%) | | 0.74 |

Additional Table ; perioperative details of uncemented and cemented hemiartroplasty
